# Integrated Genomic Analysis of Chromosomal Alterations and Mutations in B-Cell Acute Lymphoblastic Leukemia Reveals Distinct Genetic Profiles at Relapse

**DOI:** 10.3390/diagnostics10070455

**Published:** 2020-07-04

**Authors:** Maribel Forero-Castro, Adrián Montaño, Cristina Robledo, Alfonso García de Coca, José Luis Fuster, Natalia de las Heras, José Antonio Queizán, María Hernández-Sánchez, Luis A. Corchete-Sánchez, Marta Martín-Izquierdo, Jordi Ribera, José-María Ribera, Rocío Benito, Jesús M. Hernández-Rivas

**Affiliations:** 1 Escuela de Ciencias Biológicas, Universidad Pedagógica y Tecnológica de Colombia. Avenida Central del Norte 39-115, Tunja 150003, Boyacá, Colombia; maribel.forero@uptc.edu.co; 2 IBSAL, IBMCC, Universidad de Salamanca-CSIC, Cancer Research Center, Campus Miguel de Unamuno, 37007 Salamanca, Spain; adrianmo18@gmail.com (A.M.); crisrmontero@hotmail.com (C.R.); mahesa2504@hotmail.com (M.H.-S); lacorsan@hotmail.com (L.A.C.-S.); marta.martini@usal.es (M.M.-I.); 3 Servicio de Hematología, Hospital Clínico de Valladolid, Av. Ramón y Cajal, 3, 47003 Valladolid, Spain; agarciaco@saludcastillayleon.es; 4 Servicio de Oncohematología Pediátrica, Hospital Universitario Virgen de la Arrixaca, Murcia, Ctra. Madrid-Cartagena, s/n, 30120 Murcia, El Palmar, Spain; josel.fuster@carm.es; 5 Servicio de Hematología, Hospital Virgen Blanca, Altos de Nava s/n, 24071 León, Spain; ndelasheras22@hotmail.com; 6 Servicio de Hematología, Hospital General de Segovia, C/Luis Erik Clavería Neurólogo S/N, 40002 Segovia, Spain; jqueizan@saludcastillayleon.es; 7 Servicio de Hematología, Hospital Universitario de Salamanca, Paseo de San Vicente, 88-182, 37007 Salamanca, Spain; 8 Acute Lymphoblastic Leukemia Group, Josep Carreras Leukaemia Research Institute, Carretera de Canyet, s/n, Barcelona, 08916 Badalona, Spain; jribera@carrerasresearch.org; 9 Servicio de Hematología Clínica, Institut Català d’Oncologia, Hospital Germans Trias i Pujol, Josep Carreras Research Institute, Universitat Autònoma de Barcelona, Carretera de Canyet, s/n, Barcelona, 08916 Badalona, Spain; jribera@iconcologia.net; 10 Departamento de Medicina, Universidad de Salamanca, Campus Miguel de Unamuno. C/Alfonso X El Sabio s/n, 37007 Salamanca, Spain

**Keywords:** acute lymphoblastic leukemia (ALL), relapse, next-generation sequencing (NGS), array comparative genomic hybridization (aCGH), multiplex ligation-dependent probe amplification (MLPA), *IKZF1*, *TP53*

## Abstract

The clonal basis of relapse in B-cell precursor acute lymphoblastic leukemia (BCP-ALL) is complex and not fully understood. Next-generation sequencing (NGS), array comparative genomic hybridization (aCGH), and multiplex ligation-dependent probe amplification (MLPA) were carried out in matched diagnosis–relapse samples from 13 BCP-ALL patients to identify patterns of genetic evolution that could account for the phenotypic changes associated with disease relapse. The integrative genomic analysis of aCGH, MLPA and NGS revealed that 100% of the BCP-ALL patients showed at least one genetic alteration at diagnosis and relapse. In addition, there was a significant increase in the frequency of chromosomal lesions at the time of relapse (*p* = 0.019). MLPA and aCGH techniques showed that *IKZF1* was the most frequently deleted gene. *TP53* was the most frequently mutated gene at relapse. Two *TP53* mutations were detected only at relapse, whereas the three others showed an increase in their mutational burden at relapse. Clonal evolution patterns were heterogeneous, involving the acquisition, loss and maintenance of lesions at relapse. Therefore, this study provides additional evidence that BCP-ALL is a genetically dynamic disease with distinct genetic profiles at diagnosis and relapse. Integrative NGS, aCGH and MLPA analysis enables better molecular characterization of the genetic profile in BCP-ALL patients during the evolution from diagnosis to relapse.

## 1. Introduction

Acute lymphoblastic leukemia (ALL) is a disease with specific genetic alterations associated with drug resistance, treatment failure and disease relapse [1,2]. Despite vast improvements in the treatment of childhood and adult ALL in recent years, the outlook for relapsed leukemia remains poor, highlighting the need for innovative treatment approaches [3]. It is well known that relapsed ALL is a heterogeneous disease and that distinct genetic alterations may be unique to small subgroups of patients [3]. Genomic studies of matched diagnosis–relapse samples from ALL patients have shed light on the clonal evolution that leads to relapse, the pathways associated with chemoresistance, and the potential targets for therapy [4,5,6,7,8,9]. However, the mechanisms that probably fuel an ALL relapse are not fully understood. A combined analysis of gene mutations and copy number alterations (CNAs) could provide valuable insight into the discovery of the patterns of clonal evolution and the biomarkers that predict a greater likelihood of relapse in ALL [3,10,11]. Here, we have performed an integrated and sequential genomic analysis combining next-generation sequencing (NGS), array comparative genomic hybridization (aCGH), and multiplex ligation-dependent probe amplification (MLPA) to identify the clonal shifts related to ALL progression.

## 2. Materials and Methods

### 2.1. Patients

Thirteen paired diagnosis and first relapse samples of B-cell precursor acute lymphoblastic leukemia (BCP-ALL) patients (4 children and 9 adults) were eligible for this study. The patients were treated in accordance with the risk-adapted protocols of PETHEMA (Programa Español de Tratamientos en Hematología) and SEHOP (Sociedad Española de Hematología y Oncología Pediátrica). The diagnosis of ALL was based on morphological, immunophenotypic and genetic features of leukemic blast cells, as described previously [12]. The patients’ demographic information, clinical characteristics, risk classification, response to therapy and survival were recorded. The study was approved by the local ethical committee, the Comité Ético de Investigación Clínica, at the Hospital Universitario de Salamanca. Written informed consent was obtained from each patient or their legal guardian before entering the study.

Table 1 shows the characteristics of the patients included in this study. The median age was 31 years (range 4–80 years). The median percentage of blast counts in their bone marrow was 82% (range 45–96%). Fifty-four per cent of the patients had none of the chromosomal abnormalities associated with poor risk (t(9;22), t(v;11q23) or hypodiploidy). Ninety-two per cent of patients died presenting a 5-year overall survival probability of 15% (median: 22 months, 95% CI: 3.2–40.8 (Table 1 and Table S1)).

### 2.2. DNA Isolation and Next-Generation Sequencing Assay (NGS)

Genomic DNA was extracted from frozen fixed bone marrow cell samples with a QIAmp DNA Mini Kit (Qiagen, Valencia, CA, USA) following the manufacturer’s instructions. The mutational status of the *JAK2* (exons 12 to 16), *PAX5* (exons 2 and 3), *LEF1* (exons 2 and 3), *CRLF2* (exon 6), *IL7R* (exon 5) and *TP53* (exons 4–11) genes was investigated using two preconfigured 96-well primer plates (Roche, Branford, CT, USA) with titanium amplicon chemistry (454 Life Sciences, Branford, CT, USA). The above-mentioned genes were selected due to their well-defined roles as mutational hot spots in BCP-ALL [13,14,15,16,17,18,19,20,21,22,23]. A variant analysis was performed using GS Amplicon Variant Analyzer 2.5.3 (454 Life Sciences, Roche Applied Science) and Sequence Pilot version 3.4.2 (JSI Medical Systems, Kippenheim, Germany) software [24,25]. The variants were filtered to display the sequence variants occurring in more than 2% of bidirectional reads per amplicon in at least one patient [26,27,28]. All somatic mutations were searched on the online COSMIC database—Catalogue of Somatic Mutations in Cancer (http://cancer.sanger.ac.uk/cancergenome/projects/cosmic) and the IARC *TP53* database—International Agency for Research on Cancer (http://p53.iarc.fr/p53Sequences.aspx) [29]. The sequence variations identified by NGS were independently validated using conventional Sanger sequencing and/or a separate setup of the NGS re-sequencing run.

### 2.3. Oligonucleotide Array Comparative Genomic Hybridizations (Array-CGH)

All samples were tested on an aCGH 12X135K array platform (Roche NimbleGen, Madison, WI, USA). Raw log_2_ ratios were segmented using the copy number R package (version 1.20.0) [30]. We used the GISTIC algorithm—(version 2.0.23) to identify statistically significant minimal common altered regions (MCRs) and the broad CNAs present in the samples [31]. The Database of Genomic Variants from Toronto (DGV, http://dgv.tcag.ca/dgv/app/home) was used to exclude DNA variations located in regions with defined copy number variations. All CNAs with an overlap of more than 50% with respect to those reported in the DGV were excluded.

### 2.4. Multiplex Ligation-Dependent Probe Amplification (MLPA)

MLPA reactions were performed using the SALSA MLPA P335-B1 ALL-IKZF1 probemix (MRC-Holland, Amsterdam, Netherlands) according to the manufacturer’s instructions. DNA samples from three healthy donors were used as controls. The P335-B1 probemix contains probes for the following genes: *IKZF1*, *CDKN2A/B*, *PAX5*, *EBF1*, *ETV6*, *BTG1*, *RB1*, as well as genes from the X/Y PAR1 region (*CRLF2*, *CSF2RA*, *IL3RA* and *P2RY8*). MLPA amplification products were analyzed on an ABI 3130xl Genetic Analyzer (Applied Biosystems/Hitachi) with GeneMapper software V.3.7, using the Genescan 500LIZ internal size standard (Applied Biosystems). The copy number at each locus was estimated according to Schwab et al. [32].

According to the probemix contained in the P335-B1 MLPA kit, an integrative MLPA–aCGH analysis was performed to identify gene deletions in the *IKZF1*, *CDKN2A/B*, *PAX5*, *EBF1*, *ETV6*, *BTG1* and *RB1* genes, as well as genes from the X/Y *PAR1* region (*CRLF2, CSF2RA, IL3RA* and *P2RY8*). The copy number at each locus was estimated according to the method of Schwab et al. [32], whereby values above 1.3, between 1.3 and 0.75, between 0.75 and 0.25, and below 0.25 were considered as gain, normal, hemizygous loss, and homozygous loss, respectively. It was possible to distinguish the gene deletions identified either by MLPA or by aCGH analysis, or by the both methods. The distributions of the probes in each platform are illustrated in Figure S1.

### 2.5. Statistical Methods

The differences between groups were compared by the chi-square, Fisher’s exact, and Mann–Whitney tests, as appropriate. Values of *p* < 0.05 were considered to be statistically significant. Analyses were conducted using IBM SPSS version 21.0 (IBM Corp., Armonk, NY, USA).

The materials, procedures and statistical analyses are described in detail in the Supplementary File 1.

## 3. Results

### 3.1. Recurring Genomic Alterations in Matched Diagnosis–Relapse BCP-ALL Samples

**Figure 1:**
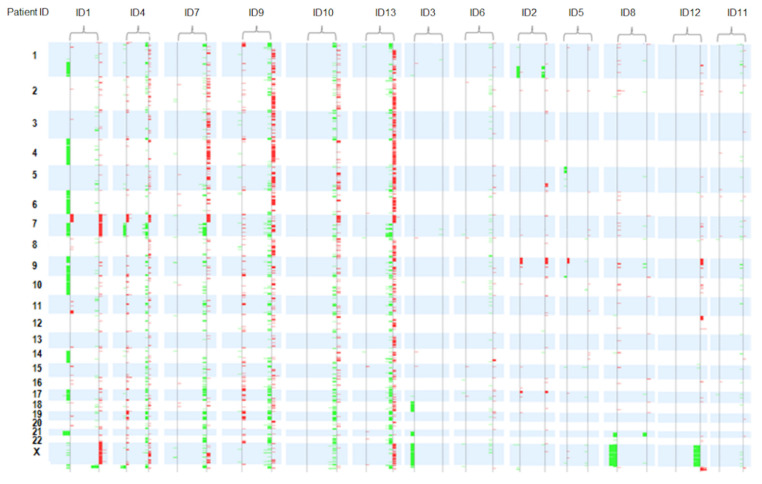
Summary of array comparative genomic hybridization results in 13 paired diagnosis and relapse samples. All patients exhibited heterogeneous changes in the pattern of copy number alterations (CNAs) from diagnosis to relapse. The paired diagnostic/relapse samples were ordered from highest to lowest number of CNAs. Losses: green. Gains: red.

**Figure 2:**
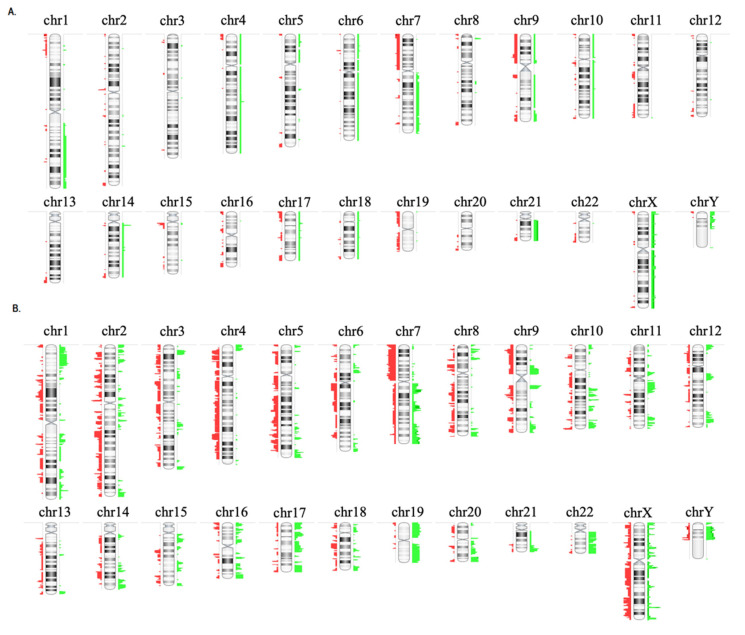
Copy number alterations observed in 13 paired diagnostic/relapse samples. (**A**) CNAs observed in all samples at diagnosis, (**B**) CNAs observed in all samples at relapse. To the right of each chromosome the regions with gains are shown in green and to the left the regions with losses are shown in red.

The aCGH detected 1451 somatic genetic lesions in 13 paired (diagnosis and relapse) BCP-ALL samples. The number of genetic lesions varied significantly between patients (1–287 lesions; median, 16 per sample). There were no significant differences in the number of CNAs between children and adults (*p* = 0.765), or between patients who had early relapses and those who did not (*p* = 0.731). Figure 1 and Figure 2 and Figure S2 show the main aCGH findings at diagnosis and relapse, with a significant increase in the number of lesions at relapse. There was a median of six alterations per sample at diagnosis and of 47 at relapse (*p* = 0.019).

Figure 1 and Figure 2 and Figure S2 show the patterns and frequencies of DNA copy alterations observed in the 13 paired diagnosis/relapse samples. The most recurrent broad and focal copy number changes observed at diagnosis and/or relapse were: dup(1q) at 23%, dup(X) at 31%, dup(21) at 15%, del(7 or 7p) at 77%, dup(7q) at 31%, del(9p) at 62%, del(12p) at 15%, del(13q) at 23% and del(17p) at 15%. Of these, the deletions located on 7p and 9p were the most frequently focal and broad chromosomal alterations detected at diagnosis and/or relapse (77% and 62%, respectively (Table S1)).

**Figure 3:**
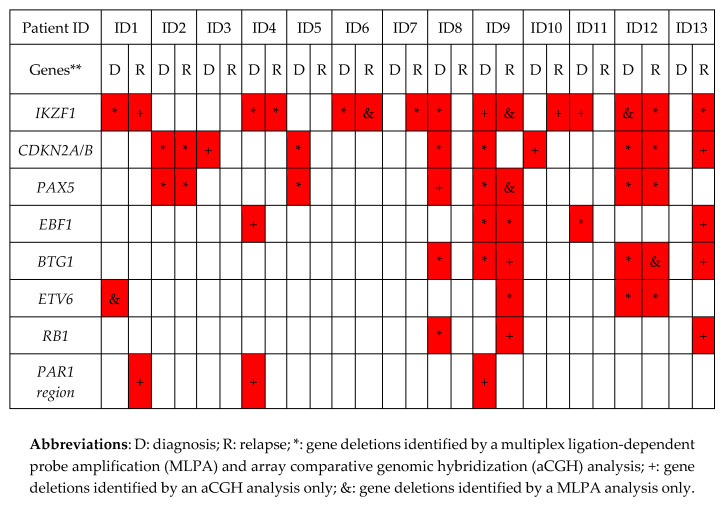
Gene deletions identified by an integrative MLPA–aCGH analysis. ** The P335-B1 probemix contains probes for the following genes: *IKZF1* (eight probes at 7p12.2), *CDKN2A/B* (three probes at 9p21.3), *PAX5* (seven probes at 9p13.2), *EBF1* (four probes at 5q33.3), *ETV6* (six probes at 12p13.2), four probes for *BTG1* and the *BTG1* downstream region (at 12q21.33), *RB1* (five probes at 13q14.2), as well as genes from the X/Y *PAR1* region (*CRLF2, CSF2RA, IL3RA* and *P2RY8* (five probes at Xp22.33)). Additionally, there is one probe at Yp11.31 (*ZFY*) and one at 9p24.1.

Our integrative MLPA–aCGH analysis showed that the percentages of deleted genes in the following paired diagnosis/relapse BCP-ALL samples were: *IKZF1*, 54% vs. 62%, *p* = 0.691; *CDKN2A/B*, 54% vs. 23%, *p* = 0.107; *PAX5*, 38% vs. 23%, *p* = 0.673; *EBF1*, 23% vs. 15%, *p* = 1.000; *BTG1,* 23% vs. 23%, *p* = 1.000; *ETV6,* 15% vs. 15%, *p* = 1.000; *RB1*, 8% vs. 15%, *p* = 1.000, and *PAR1,* 15% vs. 8%, *p* = 1.0. Thus, no statistically significant differences in the frequency of these gene deletions were found between diagnosis and relapse. Figure 3 compares the main complementary findings at the two points in disease evolution by MLPA and/or aCGH techniques.

A NGS analysis revealed six mutations in 4/13 (31%) patients (three children: ID2, ID3, ID4, and one adult: ID7)) at diagnosis and/or relapse. Notably, three of them (ID2, ID3 and ID4) did not have poor risk cytogenetics at diagnosis. Likewise, it should be mentioned that two of them were treated at diagnosis with high risk protocols (ID2 and ID7), one with an intermediate risk protocol (ID4) and one with a low risk protocol (ID3 (Table S1)). Of these six mutations, a sequence analysis revealed three missense mutations, one splicing site mutation and two deletion-insertions. *TP53* was the most frequently mutated gene (4/13, 31%), whereas *PAX5* was only mutated in one adult patient (ID7 (1/13, 8%)). Interestingly, two *TP53* mutations were only detected at relapse (ID3: c.829_842delins14 and ID7: c.-8_4del12), whereas the remaining were present from diagnosis and maintained at relapse (ID2: 817_821delinsGACCC, ID4: c.832C > T, ID7: c.818G > C (Table 2)).

The complementary integrative genomic analysis using aCGH, MLPA and NGS revealed that 100% of ALL patients showed at least one genetic alteration at diagnosis and relapse (mutation, loss and/or gain, or chromosomal rearrangement identified by Fluorescence in situ hybridization (FISH) ). The frontline risk-adapted protocols, outcome, clinical status, karyotype, FISH, NGS, aCGH and MLPA analysis of each patient are shown in Table S1.

### 3.2. Heterogeneous Patterns of Genetic Evolution in Paired Diagnosis and Relapse Samples

Tables S2–S4 show the regions of statistically significant recurrent amplification and deletion that were retained, lost, or acquired as new lesions at relapse, respectively (*q-value* < 0.05).

The statistically significant peaks retained at relapse included gains on 10q26.13 (*FAM53B)*, 15q11.2 and losses on 1p36.32, 5p15.33, 9p21.3 (*PTPLAD2, MLLT3*), 9p21.2 (*CDKN2A*, *CDKN2B*, *DMRTA1*) and 10q26.3. The significant peaks lost at relapse included losses on 8q24.3 and 19p13.3 (*TCF3, E2A*). Finally, the significant peaks acquired as new lesions at relapse included gains on 1p36, 1q21, 2p13.3 (*DYSF*), 3q21, 4p16, 5q33.1 (*PDGFRB*), 7q36.1 (*EZH*), 10q25.2, (*ADD3*), 14q32.31 (*BCL11B*), and losses on 9q34.2 and 13q34.

**Figure 4:**
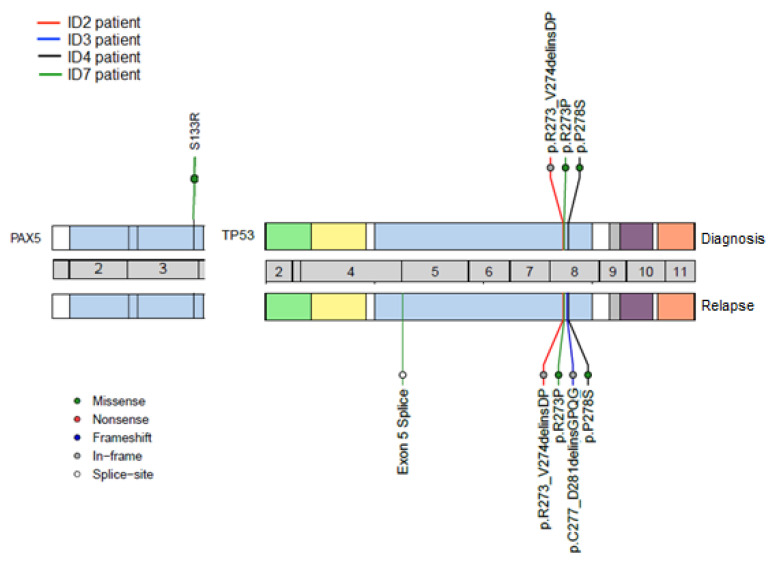
Mutations identified by next-generation sequencing (NGS) showed patterns of genetic evolution in paired diagnostic/relapse samples. Patient ID2 (red line) and Patient ID4 (black line): retained mutations in the *TP53* gene. Patient ID7 (green line): retained one mutation in the *TP53* gene, acquired a new mutation in the *TP53* gene and lost one mutation in the *PAX5* gene at relapse. Patient ID3 (blue line): acquired a new mutation in the *TP53* gene at relapse.

An integrative analysis showed that all patients exhibited heterogeneous changes in the pattern of CNAs from diagnosis to relapse, indicating that the profile of the relapse samples was genomically distinct: 8% of patients acquired only new genetic lesions at relapse, 38% of patients acquired new lesions and lost lesions present at diagnosis, and 54% of the patients simultaneously retained, lost and acquired lesions at relapse (Figure 2, Figure 3 and Figure 4).

The MLPA–aCGH analysis revealed that most of the patients (5/7, 71%) with deletions of *IKZF1* (7p) at diagnosis retained this genetic alteration at relapse (children: ID1 and ID4, and adults: ID6, ID9 and ID12). By contrast, only three (ID2, ID9, ID12) of seven patients (42.9%) with *CDKN2A/B* and/or *PAX5* deletions (9p) retained these deletions at relapse. It should be noted that the adult patient ID13, who presented an elevated number of CNAs at relapse, acquired new lesions in the chromosomal regions that harbored the *EBF1, CDKN2A/B, IKZF1, BTG1* and *RB1* genes. Finally, the losses on 17p were also identified in two patients (child ID2 and adult ID9) at diagnosis, being retained at relapse in child ID2. An insertion/deletion mutation in *TP53* was also identified in this pediatric patient at diagnosis and relapse (Tables S1 and S2, Figure 1, Figure 3 and Figure 4).

**Figure 5:**
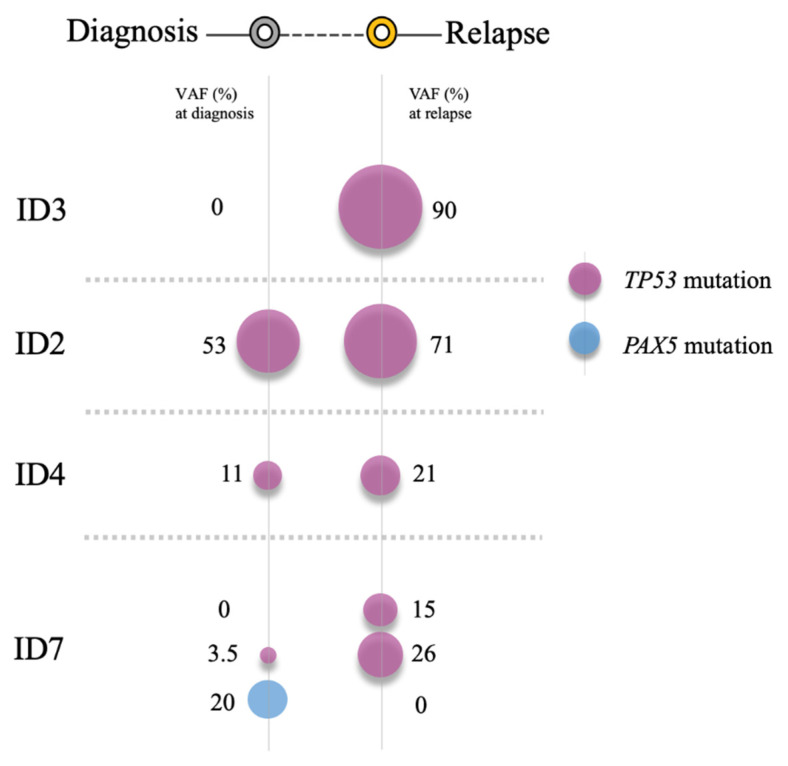
Clonal changes of mutations detected in paired diagnostic/relapse samples. Patient ID2 and Patient ID4 retained TP53 mutations, thereby increasing their mutational burden. Patient ID7 acquired a *TP53* mutation at relapse and maintained another in *TP53* from diagnosis, and showed a loss of *PAX5* mutation at relapse. Patient ID3 had acquired a *TP53* mutation by the time of relapse.

Table 2 details the mutations observed at both times and describes their mutational burden. Interestingly, two *TP53* mutations were acquired at relapse (ID3, ID7), whereas all three *TP53* mutations, which were detected from diagnosis, had an increase in their mutational burden at relapse (ID2, ID4, ID7 (Figure 5)). Thus, in the pediatric patient ID2, an increase in the *TP53* mutant clone burden was observed at relapse (53% to 71%), as well as in the pediatric patient ID4 (11% to 21%) and the adult patient ID7 (3.5% to 26%), respectively.

It is worth mentioning that the pediatric patient ID3, who was stratified as at low risk of disease at diagnosis, had acquired a mutation in *TP53* by relapse. Likewise, it should be noted that one adult patient (ID7) showed a co-occurrence of mutations in two different genes, *TP53* and *PAX5*. In the *TP53* gene, the splice mutation (c.-8_4del12) was detected only at relapse (mutational burden: 15%) whereas the missense mutation (c.818G > C) was present at both times (mutational burden: 3.5% vs. 26%). In the *PAX5* gene, only one missense mutation (c.399T > A) was observed at diagnosis (mutational burden: 20%). This patient had also acquired a deletion in the *IKZF1* gene by relapse (Table S1 and Figure 3 and Figure S3). The integrative NGS, aCGH, and MLPA analysis enabled a better molecular characterization of the genetic profile in ALL patients during the evolution of their disease from diagnosis to relapse.

## 4. Discussion

The present study, carried out in sequential ALL patients at diagnosis and relapse, showed that all 13 ALL patients exhibited heterogeneous clonal changes in terms of CNAs and mutations between diagnosis and relapse, involving the acquisition, loss and maintenance of lesions at relapse. The shared lesions between the relapse clone and the predominant clone at diagnosis suggest a common pre-leukemic origin [22], while the acquired lesions provide unequivocal evidence of a second clone that was present as a minor population at diagnosis, but acquired different genetic alterations before emerging as the relapse clone [5].

An integrated NGS, aCGH, and MLPA analysis allowed for the identification of alterations on the *IKZF1* (7p) and *TP53* (17p) genes in paired diagnostic and relapse samples; these were more frequent at relapse than at diagnosis. Both genetic events could have strongly influenced disease relapse and the short survival of these patients. In this study, *TP53* is the most frequently mutated gene at relapse (31%). *TP53* abnormalities (deletion and/or mutation) have been associated with a resistance to treatment and worse prognosis in childhood and adult ALL [8,33]. *TP53* gene abnormalities have a key role in ALL relapse, as they independently predict a high risk of treatment failure in ALL patients [8,24,34]. The presence of *TP53* alterations has been associated with a reduced response rate to induction therapy and correlated with a shortened duration of survival, even after successful reinduction therapy [35,36]. Different therapeutic strategies to target mutant p53 have been developed for the high risk *TP53*-mutant ALL, such as the use of the small molecule APR-246 which exhibits antileukemia activity in *TP53*mut BCP-ALL, targeting non-functional mutant p53 and restoring its tumor suppressive function [37].

*IKZF1* was the most frequently deleted gene, the incidence of deletions being greater at relapse than at diagnosis. Similarly, as seen in previous studies, the frequency of *IKZF1* deletions was higher in adults than in children [38]. *IKZF1* deletions have been associated with a higher risk of relapse in ALL and have been shown to be a hallmark of *BCR-ABL1*-positive ALL, although they have also been identified in a fraction of *BCR-ABL1*-negative ALL patients [10,38,39,40,41,42,43], as noted in our study. In recent years, *IKZF1* deletions and *TP53* alterations are being recognized as important markers of poor prognosis in ALL after a first relapse, mainly in children [9,39,44]. Therefore, these alterations could contribute to the re-stratification of risk for ALL patients and to proposing timely therapeutic strategies such as treatment intensification and identifying candidates for transplantation or for inclusion in clinical trials due to their high risk of suffering a second relapse [34,45,46].

In the present study, heterogeneous patterns of genetic evolution in paired diagnostic and relapse samples were observed that are consistent with those reported in previous studies [47]. In particular, two *TP53* mutations were only detected at relapse (patients ID3 and ID7), whereas all three *TP53* mutations increased their mutational burden between diagnosis and relapse (patients ID2, ID4 and ID7). It should be noted that two of the six *TP53* mutations identified had not been reported in the genomic databases.

ALL is clonally heterogeneous and genetic lesions in minor clones may confer resistance to therapy and promote disease relapse (e.g., *TP53*, *IKZF1*, *CREBBP*) [22]. The low proportion of the minor relapse subclone at diagnosis suggests that the leukemia at diagnosis contains genetically diverse subclones. Therapy would aim to select the eventual dominant relapse clone whose alterations confer resistance to treatment [48]. Thus, *TP53* mutations could be considered as driver mutations that probably confer a selective growth advantage on ALL tumor cells at relapse [24]. Preclinical studies and clinical experience have shown that leukemic blasts are more resistant at relapse than at diagnosis. Mechanisms of resistance may include the selection of a pre-existing resistant subclone or the acquisition of additional genomic lesions under the selective pressure of chemotherapy, as observed in our study [4].

## 5. Conclusions

In summary, the present study provides additional evidence that the clonality of ALL is genetically dynamic from diagnosis to relapse. The integrative NGS, aCGH, and MLPA analysis enabled a better molecular characterization of the genetic profile in ALL patients during the evolution of their disease, showing distinct genetic profiles at diagnosis and relapse. With this study, the utility of simultaneously identifying CNAs and mutations at the time of diagnosis and relapse was evidenced, which is clinically important to predict the evolution of the patients. New genomic strategies to identify various genetic lesions from a single sample and in a single experiment are currently being solved by designing specific panels for each type of hematological disease, which contribute to the improvement of the risk stratification, promoting the use of personalized treatment in ALL.

## Figures and Tables

**Table 1:** Characteristics of the patients with B-cell precursor acute lymphoblastic leukemia (BCP-ALL) included in the study.

Characteristics	Patients (n = 13)
Age at diagnosis (years), median (range)	31 (4–80)
Male/Female, (%)	3/10 (23.1/76.9)
Bone marrow blast ^1^, median (range)	82 (45–96)
White blood cell count (×109/L), median (range)	27 (3–168)
Hb count (g/L), median (range)	105 (39–160)
Platelet count (×109/L), median (range)	73 (29–248)
Elevated LDH, (U/L) level, (%)	66.7
Cytogenetics	
Poor risk ^2^ (%)	46.2
Others (%)	53.8
Risk group ^3^	
Low risk (%)	7.7
Intermediate risk (%)	23.1
High risk (%)	69.2
Time to relapse	
Very early relapse ^4^ (%)	53.8
Early relapse ^4^ (%)	15.4
Late relapse ^4^ (%)	30.8
5-year overall survival probability % (median, 95% CI)	15.3 (22, 3.2–40.8)

^1^ Estimated by flow cytometry. ^2^ Includes the unfavorable abnormalities t(9;22), t(v;11q23) and hypodiploidy. ^3^ Risk group stratification was mainly designated according to the Programa Español de Tratamientos en Hematología (PETHEMA) protocols. ^4^ Time of relapse criteria: very early, earlier than 18 months after initial diagnosis and less than 6 months after the cessation of frontline treatment; early, more than 18 months after initial diagnosis, but less than 6 months after the cessation of frontline treatment; late, more than 6 months after the cessation of frontline treatment.

**Table 2:** Description of somatic mutations observed in diagnosis–relapse BCP-ALL patients. All three TP53 mutations retained at relapse increased their mutational burden to relapse (c.818G > C from 3.5% to 26%, c.832C > T from 11% to 21% and 817_821delinsGACCC from 53% to 71%).

Patient ID	Gene	Type of Mutation	Mutation	AA Change	Database	Moment	Mutational Burden
ID2	*TP53-E08*	Indel	c.817_821delinsGACCC	p.R273_V274delinsDP	Undescribed	Diagnosis	53%
						Relapse	71%
ID4	*TP53-E08*	Missense	c.832C > T	p.P278S	COSM10939/*TP53* website http://p53.fr/	Diagnosis	11%
						Relapse	21%
ID7	*PAX5-E03*	Missense	c.399T > A	p.S133R	Undescribed	Diagnosis only	20%
	*TP53-E08*	Missense	c.818G > C	p.R273P	COSM165077/*TP53* website http://p53.fr	Diagnosis	3.5%
						Relapse	26%
	*TP53-E05*	Splicing	c.-8_4del12	Splice_Intron 5 SA	*TP53* website http://p53.fr	Relapse only	15%
ID3	*TP53-E05*	Missense	c.829_842delins14	p.C277_D281delinsGPQG	Undescribed	Relapse only	90%

## Supplementary Materials

The following are available online at https://www.mdpi.com/2075-4418/10/7/455/s1. All data analyzed during this study are included in this published article and its Supplementary File 1 which contains: Table S1. Frontline risk-adapted protocols, outcome, clinical status, karyotype, FISH, NGS, aCGH and MLPA analysis in matched diagnosis-relapse BCP-ALL patients; Table S2. Description of somatic mutations observed in diagnosis–relapse BCP-ALL patients; Table S3. Regions of significant recurrent amplification and deletion retained at relapse (*q-value* < 0.05); Table S4. Regions of significant recurrent deletion lost at relapse (*q-value <* 0.05); Table S5. Regions of significant recurrent amplification and deletion acquired at relapse (*q-value* < 0.05); Figure S1. Location of the probes in the X/Y PAR1 region provided in the MLPA probemix and NimbleGen high-density microarray platform; Figure S2. Patterns and frequencies of DNA copy alterations observed in 13 paired diagnostic/relapse samples; Figure S3. Gene deletions identified by integrative MLPA-aCGH analysis and Figure S4. Mutations identified by NGS showed patterns of genetic evolution in paired diagnostic/relapse samples. The datasets generated during the current study are available in the GEO Accession viewer repository (GSE12803), LINK: [https://www.ncbi.nlm.nih.gov/geo/query/acc.cgi?acc=GSE128031].
