# Supplementary material for: Integrated Genomic Analysis of Chromosomal Alterations and Mutations in B-Cell Acute Lymphoblastic Leukemia Reveals Distinct Genetic Profiles at Relapse
Source: Diagnostics (Basel). 2020 Jul 4;10(7):455. doi: 10.3390/diagnostics10070455 (PMC7400270; doi:10.3390/diagnostics10070455)
Supplement: Supplementary file 1 [file diagnostics-10-00455-s001.pdf]

Supplementary File 1

Article

Integrated genomic analysis of chromosomal alterations and mutations in B-cell acute lymphoblastic leukemia reveals distinct genetic profiles at relapse

Maribel Forero-Castro [1†], Adrián Montaño [2†], Cristina Robledo [2], Alfonso García de Coca [3], José Luis Fuster [4], Natalia de las Heras [5], José Antonio Queizán [6], María Hernández-Sánchez [2], Luis A. Corchete-Sánchez [2,7], Marta Martín-Izquierdo [2], Jordi Ribera [8], José-María Ribera [9], Rocío Benito [2*], Jesús M. Hernández-Rivas [2,7,10*]

[1]Escuela de Ciencias Biológicas, Universidad Pedagógica y Tecnológica de Colombia. Avenida Central del Norte 39-115, 150003, Tunja, Boyacá, Colombia; maribel.forero@uptc.edu.co

[2]IBSAL, IBMCC, Universidad de Salamanca-CSIC, Cancer Research Center, Campus Miguel de Unamuno, 37007 Salamanca, Spain; adrianmo18@gmail.com (A.M.); crisrmontero@hotmail.com (C.R.); mahesa2504@hotmail.com (M.H.S); lacorsan@hotmail.com (L.A.C.S); marta.martini@usal.es (M.M.I); beniroc@usal.es (R.B.); jmhr@usal.es (J.M.H.R)

[3]Servicio de Hematología, Hospital Clínico de Valladolid, Av. Ramón y Cajal, 3, 47003 Valladolid, Spain; agarciaco@saludcastillayleon.es

[4]Servicio de Oncohematología Pediátrica, Hospital Universitario Virgen de la Arrixaca, Murcia, Ctra. Madrid-Cartagena, s/n, 30120 El Palmar, Murcia, Spain; josel.fuster@carm.es

[5]Servicio de Hematología, Hospital Virgen Blanca, Altos de Nava s/n, 24071 León, Spain; ndelasheras22@hotmail.com

[6]Servicio de Hematología, Hospital General de Segovia, C/ Luis Erik Clavería Neurólogo S/N, 40002 Segovia, Spain; jqueizan@saludcastillayleon.es

[7]Servicio de Hematología, Hospital Universitario de Salamanca, Paseo de San Vicente, 88-182, 37007 Salamanca, Spain; lacorsan@hotmail.com (L.A.C.S); jmhr@usal.es (J.M.H.R)

[8]Acute Lymphoblastic Leukemia Group, Josep Carreras Leukaemia Research Institute, Carretera de Canyet, s/n, 08916 Badalona, Barcelona, Spain; jribera@carrerasresearch.org

[9]Servicio de Hematología Clínica, Institut Català d'Oncologia, Hospital Germans Trias i Pujol, Josep Carreras Research Institute, Universitat Autònoma de Barcelona, Carretera de Canyet, s/n, 08916 Badalona, Barcelona, Spain; jribera@iconcologia.net

[10]Departamento de Medicina, Universidad de Salamanca, Campus Miguel de Unamuno. C/ Alfonso X El Sabio s/n. 37007-Salamanca; jmhr@usal.es (J.M.H.R)

† M.F.-C. and A.M. contributed equally to this work.

*Sharing senior authorship

*Correspondence: jmhr@usal.es (J.M.H.R.) and beniroc@usal.es (R.B.); Tel.: Phone: + 34 923291384 (J.M.H.R.) and + 34 923294812 (R.B.).

Table of contents of supplementary patients and methods

DNA isolation …………………………………………………………………………….. ………..3

Oligonucleotide array comparative genomic hybridizations……………………………………..3

Array-CGH data analysis methods……………………………………………………….……......3

Amplicon library preparation………………………………..…………………………..……..3

Amplicon library pooling and purification……………….……………………………............4

Emulsion PCR and sequencing………………………………………………………………...4

Data processing and analysis……………………..4

MLPA…………………………………………………...4

List of supplementary tables

Table S1. Frontline risk-adapted protocols, outcome, clinical status, karyotype, FISH, NGS,

aCGH and MLPA analysis in matched diagnosis-relapse BCP-ALL patients.…………….……..5

Table S2. Regions of significant recurrent amplification and deletion retained at relapse (*q-value*<0.05)……………………………………………………………………………………………9

Table S3. Regions of significant recurrent deletion lost at relapse (*q-value* <0.05)…… …………...10

Table S4. Regions of significant recurrent amplification and deletion acquired at relapse (*q-value* <0.05)………………………………………………………………………………...……………11

List of supplementary figures

Figure S1. Location of the probes in the X/Y PAR1 region provided in the MLPA probemix and NimbleGen high-density microarray platform …………………………………………….........13

Figure S2. Patterns and frequencies of DNA copy alterations observed in 13 paired diagnostic/relapse samples…………………………………………………………………………………………….14

References for supplementary information

Supplementary patients and methods

DNA isolation

Genomic DNA was extracted from frozen bone marrow or peripheral blood fixed cell samples using a QIAamp DNA Mini Kit (Qiagen, Valencia, CA, USA) following the manufacturer's instructions. DNA quality was assessed as the A260/A280 ratio with a NanoDrop ND-1000 spectrophotometer (NanoDrop Technologies, Wilmington, DE, USA) and by 1% agarose gel electrophoresis. A260/A280 ≥ 1.8 and A260/A230 ≥ 1.9 ratios were required for optimal labeling of DNA samples.

Oligonucleotide array comparative genomic hybridizations

In brief, patient DNA and normal control DNA (Human Genomic DNA: Male/Female, Promega, Madison, WI, USA) samples were denatured and labeled in parallel with Cy3 for the test group and Cy5 for the control group, each through a random priming method, using Klenow fragments (NimbleGen Dual-Color DNA Labeling Kit, Roche NimbleGen, Inc., Madison, WI, USA). Following cleanup and quantification, the test and sex-matched reference DNA samples were combined in equimolar amounts and loaded into one of the twelve filled ports on the microarray slide. Hybridization was carried out in a NimbleGen Hybridization Chamber for 16-20 hours at 42°C. Subsequently, the microarray slide was washed, dried and scanned at 2-μm resolution using a NimbleGen MS 200 microarray scanner. To avoid slide batch spotting bias, samples were hybridized in random order.

Array-CGH data analysis methods

Array image files (532.tif and 635.tif) generated by the MS 200 Data Collection Software were imported into Nexus Copy Number software (version 4.1) (Biodiscovery, Inc., Hawthorne, CA, USA) for analysis. $\log_2$ values of the raw data were normalized using the loess algorithm implemented in the affy R package (version 1.50.0). Quality control measures involved checking the consistency of signal distributions across samples; unsupervised clustering was performed to check outlier samples using SIMFIT statistical software (www.simfit.org.uk) (data not shown). Each genomic region exhibiting a copy number change was examined using the University of California at Santa Cruz Genome Browser (http://genome.cse.ucsc.edu) tool to determine the location and significance of the change. Normalized $\log_2$ ratios were processed for outlier removal though winsorization and segmented by piecewise constant segmentation (PCF) using the copynumber R package (version 1.20.0). Statistically significant regions in common between cases were assessed by Genomic Identification of Significant Targets in Cancer (GISTIC) analysis [1] with a confidence level of 0.90. The statistical significance of the aberrations was displayed as the FDR (false-discovery rate) q-values obtained for each region. The method accounts for multiple-hypothesis testing using the FDR framework and assigns a value of q to each result, reflecting the probability that the event is due to chance [1]. Values of $q < 0.05$ were considered to represent statistically significant amplification and deletion peaks in children and adult patients. The Database of Genomic Variants from Toronto (DGV, http://dgv.tcag.ca/dgv/app/home) was used to exclude DNA variations located in regions with defined copy number variations. Thus, all copy number changes with more than 50% overlap with respect to those reported in DGV were excluded. Sex chromosomes were included from array-CGH data analysis. CNAs ≥ 0.5 Mb and detected by at least five consecutive aCGH probes were retained for copy number analysis. Large-scale or broad copy number alterations corresponded to regions larger than 50% of a chromosome arm. All genome-based data reported in this manuscript correspond to NCBI build 36 (hg18- Mar. 2006).

Amplicon library preparation

A sensitive next-generation amplicon deep-sequencing assay (NGS) was applied, using the Titanium amplicon chemistry (454 Life Sciences, Branford, CT, USA). For this approach, two preconfigured 96-well primer plates containing lyophilized primer pairs (Roche, Branford, CT, USA) were used to prepare the amplicon library following the procedures used in the IRON-II Study (European Leukemia Network group). The first PCR plate (termed B-ALL SeqPlate) was designed to amplify *JAK2* (exons 12 to 16), *PAX5* (exons 2 and 3), *LEF1* (exons 2 and 3), *CRLF2* (exon 6) and *IL7R* (exon 5) genes, while the second plate (*TP53* SeqPlate) was designed to amplify the *TP53* (exons 4 to E11) gene. Thus, in total, 19 amplicon preparations across 26 samples, involving 494 individual PCR reactions, were carried out. The B-ALL SeqPlate was used to generate 11 amplicons from up to eight individual samples while the *TP53* SeqPlate was used to generate eight amplicons from up to 11 individual samples, thus 88 amplicons per run were obtained of each plate. In *TP53* SeqPlate the size range of the amplified products was 404-431bp including the adaptor sequences, while in the B-ALL SeqPlate the size range was 304-427bp. The above-mentioned genes were selected due to their well-defined role as mutational hot spots in BCP-ALL [2-12].

Amplicon library pooling and purification

After generating the amplicons, amplicon library pooling in equivalent amounts was carried out. The pooled libraries could be either different amplicons of the same sample, of the same amplicon in different samples, or any combination of these. The pooled libraries were purified with Agencourt AMPure magnetic beads (Beckman Coulter, Krefeld, Germany) in order to remove small amplicons (< 100 bp) and separating only those amplicons with optimal size for high-throughput sequencing. The purified amplicon pooling was quantified by dsDNA HS Qubit® Fluorometric Quantitation Assay Kit (Life Technologies). The quality of the amplicon pool was evaluated with an Agilent 2100 Bioanalyzer.

Emulsion PCR and sequencing

The amplicon library pooling was subsequently diluted to a concentration of $2x10^6$ molecules per µl and subjected to emulsion PCR using GS Junior Titanium emPCR Kit (Lib-A) (Roche Applied Science). Forward (A beads) and reverse (B beads) reactions were carried out using 2 000 000 beads per emulsion oil tube. The copy per bead ratio used was 1:1. The diluted PCR amplicons were mixed with beads under conditions that favored one fragment per bead. The amplification reaction, breaking of the emulsions and enrichment of beads carrying amplified DNA was performed using the workflow as recommended by the manufacturer (Roche Applied Science, M Penzberg, Germany). The bidirectional sequencing was carried out for 200 cycles using full processing mode for amplicons on a GS Junior platform (454 Life Sciences, Branford, CT, USA).

Data processing and analysis

Sequencing reads in SFF (standard flowgram format) file format obtained from the 454 GS Junior sequencing run were analyzed using the GS Variant Analyzer Software 2.5.3 (454 Life Sciences, Roche Applied Science) and Sequence Pilot version 3.4.2 (JSI Medical Systems, Kippenheim, Germany) software. Sequence alignment and variant detection were performed using the following reference sequences: *JAK2:* Transcript-ID: ENST00000381652, *PAX5*:ENST00000358127, *LEF1*:ENST00000265165,*CRLF2*:ENST00000400841, *IL7R:* ENST00000303115 and *TP53*: ENST00000269305. Quality control (QC) was included to provide coverage of more than 140 reads per amplicon (70 minimum reads in both forward and reverse directions). The variants were filtered to display sequence variants occurring in more than 2% of bidirectional reads per amplicon in at least one patient [13-15]. All somatic mutations were searched in the online COSMIC database (http://cancer.sanger.ac.uk/cancergenome/projects/cosmic) and the IARC *TP53* database (http://p53.iarc.fr/p53Sequences.aspx) [16]. Variants previously reported as germline polymorphisms in the Single Nucleotide Polymorphism database (dbSNP build 138) were excluded. Sequence variations identified by NGS were independently validated using conventional Sanger sequencing from a second PCR using the original DNA and/or a separate setup of NGS PCR, emPCR and re-sequencing run.

MLPA

Variable quantities of sample DNA (50-250 ng) were subjected to MLPA reactions using SALSA MLPA P335-B1 ALL-IKZF1 probemix (MRC-Holland, Amsterdam, Netherlands) according to the manufacturer's instructions. DNA from three healthy donors was used as control samples. The P335-B1 probemix contains probes for the following genes: *IKZF1* (8 probes at 7p12.2), *CDKN2A/B* (3 probes at 9p21.3), *PAX5* (7 probes at 9p13.2), *EBF1* (4 probes at 5q33.3), *ETV6* (6 probes at 12p13.2), 4 probes for *BTG1* and the *BTG1* downstream region (at 12q21.33), *RB1* (5 probes at 13q14.2), as well as genes from the X/Y PAR1 region (*CRLF2, CSF2RA, IL3RA* and *P2RY8*) (5 probes at Xp22.33). Additionally, one probe at Yp11.31 (*ZFY*) and one at 9p24.1 (*JAK2*) were included to facilitate the determination of the extent of a deletion/duplication detected in patient samples. Finally, 13 reference probes were included targeting chromosomal regions that are relatively stable in ALL. The design of this MLPA-kit allows the identification of deletions and duplications of one or more chromosomal regions in each DNA sample. MLPA amplification products were analyzed on a ABI 3130xl Genetic Analyzer (Applied Biosystems/Hitachi) with the GeneMapper software V.3.7, using the Genescan 500LIZ internal size standard (Applied Biosystems). Each peak in the electropherogram corresponded to the amplification product of a specific amplicon. Each patient's electropherogram was compared with three controls. Coffalyzer MLPA DAT (MRC-Holland) software was used to analyze MLPA data. The copy number at each locus was estimated according to method of Schwab et al. [17], whereby values above 1.3, between 1.3 and 0.75, between 0.75 and 0.25, and below 0.25 were considered as gain, normal, hemizygous loss, and homozygous loss, respectively.

Supplementary tables

Table S1. Frontline risk-adapted protocols, outcome, clinical status, karyotype, FISH, NGS, aCGH and MLPA analysis in matched diagnosis-relapse B-ALL patients.

Patient ID	Sex/ age[1] (years)	Moment evaluated	aCGH findings			Gene deletions (MLPA-aCGH)	Positive *NGS* results	Genetic subtype at diagnosis	Karyotype	Positive *FISH* results	Positive molecular biology results	Risk group	Frontline therapy	Outcome	Clinical status
			aCGH[2]	Broad gains (≥5Mb)	Broad losses (≥5Mb)										
ID1	F/4	Diagnosis	A	1q<br>4<br>6<br>7q*<br>9<br>10<br>14<br>17<br>21	7p*	*IKZF1, ETV6*		Normal	46,XX[10]	N	ND	IR	PETHEMA LAL-RI/96	CR	Rel-D
		Relapse	A		7<br>X	*IKZF1, PAR1*		NA	54,XX,+X,5,+17,+21,+4mar[4]/46,XX[6]	N	UNKN	NA		NA	
ID2	F/12	Diagnosis	A	1q	9p<br>17p	*CDKN2A/B, PAX5*	*TP53*mut	*TCF3(E2A)-PBX1*	46,XX[15]	*TCF3* loss-89%<br>*TCF3/PBX1* 89%	ND	HR	PETHEMA LAL-AR/2003	CR	Rel-D

		Relapse	A	1q	9p<br>17p	*CDKN2A/B, PAX5*	*TP53*mut	*NA*	F	*TCF3/PBX1* 75%	UNKN	NA		NA	
ID3	F/14	Diagnosis	A	18<br>21<br>X		*CDKN2A/B*		Hyper (47-50)	F	*RUNX1* gain-85%	UNKN	LR	PETHEMA LAL-BR-01	CR	Rel-D
		Relapse	N				*TP53*mut	*NA*	F	*ETV6* loss-62%<br>*KMT2A(MLL)* loss-82%<br>*ABL* loss-88%<br>*BCR* loss-88%	UNKN	NA		NA	
ID4	F/15	Diagnosis	A	7q**	7p**<br>19	*EBF1, IKZF1, PAR1*	*TP53*mut	Others	46,XX,t(7;15)(p13;q12)[3] /46,XX[7]	N	ND	IR	PETHEMA LAL-RI/96	CR	Rel-D
		Relapse	A	7q*<br>19<br>22	7p*	*IKZF1*	*TP53*mut	*NA*	47,XX,der(7)t(7;?)(p13;?),+mar[10]/46,XX[7]	N	UNKN	NA		NA	
ID5	F/23	Diagnosis	A	5p<br>9q***	9p***	*CDKN2A/B, PAX5*		Others	46,XX,del(1)(q21),i(9)(q10)[15] /46,XX[5]	*ABL* gain-29%	N	IR	PETHEMA LAL-RI/2008	CR	Rel-CR-A
		Relapse	A	9p21	14q23.3			*NA*	ND	ND	UNKN	NA		NA	
ID6	M/29	Diagnosis	A		7p12.2****	*IKZF1*		*BCR-ABL1*	46,XY,t(9;22)(q34;q11)[10]	*BCR/ABL* 91%	Major BCR/ABL Positive	HR	PETHEMA LAL-Ph/2000/Imatinib	CR	Rel-D
		Relapse	A	Focal CNAs	Focal CNAs	*IKZF1*		*NA*	F	*BCR/ABL* 51%	UNKN	NA		NA	
ID7	F/31	Diagno	A	Focal	Focal		*TP53*mut/	*KMT2A(ML*	46,XX,t(4;11)(q21;q2	N	UNKN	HR	GMALL	CR	Rel-D

		sis		CNAs	CNAs		*PAX5*mut	*L)-R*	3)[10]						
		Relapse	A	7q* 17 19 22	4 7p*	*IKZF1*	*TP53*mut/ *TP53*mut	*NA*	F	*KMT2A(MLL)-R* 90%	UNKN	NA		NA	
ID8	M/32	Diagnosis	A	21 X		*CDKN2A/B, PAX5, IKZF1, BTG1, RB1*		Normal	46,XY[19]	N	ND	HR	FLAGIDA	PR (CR later on)	Rel-CR-D
		Relapse	A	21				*NA*	46,XY[10]/48,XY,+7,+21[2]	ND	UNKN	NA		NA	
ID9	F/34	Diagnosis	A		17 19	*EBF1, CDKN2A/B, PAX5, IKZF1, BTG1, PAR1*		*BCR-ABL1*	46,XX,t(9;22)(q34;q11)[8]/46,XX[2]	*BCR/ABL* 82%	Minor BCR/ABL Positive	HR	PETHEMA LAL-Ph/2000/Imatinib	PR (CR later on)	Rel-D
		Relapse	A	22	4	*EBF1, PAX5, IKZF1, ETV6, BTG1, RB1*		*NA*	F	*BCR/ABL* 81%	UNKN	NA		NA	
ID10	M/36	Diagnosis	A		9p21.3*****	*CDKN2A/B*		Hypo (<44)	34-44,XY[8]/46,XY[9]	ND	UNKN	HR	PETHEMA LAL-AR/93	PR (CR later on)	Rel-D
		Relapse	A	19 22	7p	*IKZF1*		*NA*	48,XY,+3,del(5)(p13p15),+18[8]/46,XY[11]	ND	UNKN	NA		NA	

				X											
ID11	F/38	Diagnosis	A		7p12.2****	*EBF1, IKZF1*		Hyper (47-50)	46,XX[10]	*ABL* gain-30%	N	HR	PETHEMA LAL-AR/2003	CR	Rel-CR-D
		Relapse	A	1q21	7p15			*NA*	ND	N	UNKN	NA		NA	
ID12	F/52	Diagnosis	A		9p	*CDKN2A/B, PAX5, IKZF1, ETV6, BTG1*		Hypo (<44)	39-42,XX,add(12)(p13)[5]/46,XX,add(12)(p13)[2]/46,XX[9]	ND	ND	HR	UNKN	CR	Rel-D
		Relapse	A	X	9p<br>12p	*CDKN2A/B, PAX5, IKZF1, ETV6, BTG1*		*NA*	ND	*BCR/ABL* 94%	ND	NA		NA	
ID13	F/80	Diagnosis	N					*KMT2A(MLL)-R*	46,XX[15]	*KMT2A(MLL)-R* 93%	UNKN	HR	PETHEMA LAL/OLD-FRA	CR	Rel-D
		Relapse	A	7q*<br>19<br>22	4<br>7p*<br>13<br>X	*EBF1, CDKN2A/B, IKZF1, BTG1, RB1*		*NA*	ND	ND	UNKN	NA		NA	

[1]At diagnosis. [2]Not all CNAs <0.5 Mb are shown in this table. *Consistent with an isochromosome 7q, i(7q). **Consistent with pseudodiploid karyotype with unbalanced translocation involving gain of 7q and loss of 7p. ***Consistent with an isochromosome 9q, i(9q). **** Focal loss (CNA <0.5 Mb). **Abbreviations**: A: aCGH altered, N: aCGH normal, UNKN: unknown, NA: not applicable, CR: complete remission, D: dead, LR: low risk, IR: intermediate risk, HR: high risk, ND: Not done, Hyper (47-50), low hyperdiploid (47-50 chromosomes), Hypo (<44), FISH, fluorescence in situ hybridization; F: failed, NA: not applicable.

Table S2. Regions of significant recurrent amplification and deletion retained at relapse (*q-value* <0.05). This table shows the MCR identified at diagnosis (part a) that were retained at relapse (part b).

a) **Diagnosis MCRs**							
**MCR**	**Cytoband**	**Wide peak limits**	**Probes**	**q values**	**Frequency**	**Overlap_CNV**	**Genes associated with BCP-ALL**
Amplification Peak 1	10q26.13	chr10:125287308-125627394	13	0.0029798	69.2	49.9	
Amplification Peak 2	15q11.2	chr15:1-20387217	30	0.00152	30.8	10.4	
Deletion Peak 1	1p36.32	chr1:1-3740335	82	0.043017	30.8	61.9	
Deletion Peak 2	5p15.33	chr5:1-2809848	80	0.019729	23.1	43.0	
Deletion Peak 3	9p21.3	chr9:19775843-21252803	55	0.0011035	53.8	42.7	*PTPLAD2, MLLT3*
Deletion Peak 4	9p21.2	chr9:21943224-32382231	306	0.04103	38.5	38.8	*CDKN2B, CDKN2A, DMRTA1*
Deletion Peak 5	10q26.3	chr10:133639221-135374737	57	0.039672	23.1	61.2	
b) **Relapse MCRs**							
**MCR**	**Cytoband**	**Wide peak limits**	**Probes**	**q values**	**Frequency**	**Overlap_CNV**	**Genes associated with BCP-ALL**
Amplification Peak 1	10q26.13	chr10:125596091-126882537	71	0.016885	46.2	24.1	*FAM53B*
Amplification Peak 2	15q11.2	chr15:1-20387217	30	0.016885	30.8	10.4	
Deletion Peak 1	1p36.33	chr1:1804656-3559849	38	0.0010206	38.5	41.6	
Deletion Peak 2	5p15.33	chr5:1-2809848	80	0.0015214	38.5	43.0	
Deletion Peak 3	9p21.3	chr9:19775843-22441867	95	0.00015622	46.2	68.3	*CDKN2A, IFN, MLLT3, PTPLAD2, MTAP, DCKN2B, DMRTA1*
Deletion Peak 5	10q26.3	chr10:133821131-135374737	54	0.000211	53.8	68.4	

Table S3. Regions of significant recurrent deletion lost at relapse (*q-value* < 0.05).

MCR	Cytoband	Wide peak limits	Probes	q values	Frequency	Overlap_CNV	Genes associated with BCP-ALL
Deletion Peak 1	8q24.3	chr8:142063749-146274826	130	0.04103	23.0769231	33.8	
Deletion Peak 2	19p13.3	chr19:1-5585748	160	0.04103	30.7692308	36.8	*TCF3, E2A*

Table S4. Regions of significant recurrent amplification and deletion acquired at relapse (*q-value* < 0.05).

MCR	Cytoband	Wide peak limits	Probes	q values	Frequency	Overlap_CNV	Genes associated with BCP-ALL
Amplification Peak 1	1p36.22	chr1:8732967-9911979	49	0.016885	46.2	27	
Amplification Peak 2	1p36.22	chr1:11285557-12240632	39	0.016885	53.8	17	
Amplification Peak 3	1p36.13	chr1:14909013-18795097	196	0.045783	53.8	23	
Amplification Peak 4	1q21.1	chr1:120280136-144113553	86	0.016885	38.5	6	
Amplification Peak 5	1q23.2	chr1:158012327-158639152	42	0.032569	46.2	28	
Amplification Peak 6	2p24.3	chr2:15588842-16322114	34	0.016885	46.2	32	
Amplification Peak 7	2p23.3	chr2:26490879-27444893	44	0.016885	53.8	13	
Amplification Peak 8	2p13.3	chr2:69882623-72233418	112	0.024079	38.5	24	*DYSF*
Amplification Peak 9	2p11.2	chr2:85070707-85828746	44	0.016885	46.2	23	
Amplification Peak 10	2q13	chr2:108663875-109679153	47	0.016885	46.2	36	
Amplification Peak 11	2q14.2	chr2:120938348-121845814	60	0.016885	46.2	23	
Amplification Peak 12	3p25.1	chr3:13051957-13690735	25	0.016885	46.2	29	
Amplification Peak 13	3p22.1	chr3:42663872-43126699	35	0.016885	46.2	40	
Amplification Peak 14	3q21.1	chr3:124029509-124945325	63	0.016885	46.2	24	
Amplification Peak 15	3q21.3	chr3:127842173-130850454	151	0.016885	46.2	21	
Amplification Peak 16	4p16.1	chr4:5720583-7875973	106	0.016885	46.2	34	
Amplification Peak 17	5p15.1	chr5:16664587-31286811	323	0.045783	46.2	16	
Amplification Peak 18	5q31.1	chr5:133804535-134997254	66	0.016885	53.8	18	
Amplification Peak 19	5q31.3	chr5:140903053-142072612	89	0.019505	46.2	19	
Amplification Peak 20	5q33.1	chr5:149355702-150078235	38	0.016885	46.2	9	*PDGFRB*
Amplification Peak 21	5q35.1	chr5:167592461-168387411	92	0.045783	46.2	6	
Amplification Peak 22	5q35.1	chr5:171673733-172302393	41	0.016885	46.2	42	
Amplification Peak 23	6p25.3	chr6:1-1931210	121	0.032569	46.2	43	
Amplification Peak 24	6p21.1	chr6:43423545-44935974	78	0.019505	46.2	11	
Amplification Peak 25	7q22.1	chr7:100780141-101605221	61	0.016885	46.2	40	
Amplification Peak 26	7q22.2	chr7:104673282-105652483	63	0.045783	46.2	44	

Amplification Peak 27	7q32.3	chr7:131338179-131860908	54	0.024079	38.5	29	
Amplification Peak 28	7q36.1	chr7:148179407-148764908	28	0.019505	38.5	17	*EZH2*
Amplification Peak 29	8p21.3	chr8:21548589-23589751	121	0.016885	46.2	43	
Amplification Peak 30	8q24.22	chr8:133912051-134874122	63	0.045783	38.5	12	
Amplification Peak 31	8q24.3	chr8:140615440-141545165	56	0.016885	46.2	49	
Amplification Peak 32	9p13.1	chr9:38917771-70176099	315	0.016885	76.9	26	
Amplification Peak 33	9q22.2	chr9:91245647-91827350	32	0.016885	46.2	49	
Amplification Peak 34	9q32	chr9:114501990-116298659	113	0.032569	46.2	30	
Amplification Peak 35	9q33.3	chr9:127799231-128430649	53	0.016885	46.2	27	
Amplification Peak 36	10q23.2	chr10:87804465-88565173	33	0.016885	53.8	40	
Amplification Peak 37	10q24.1	chr10:98172138-100219092	109	0.016885	46.2	10	
Amplification Peak 38	10q25.2	chr10:111864227-112971515	62	0.045783	46.2	30	*ADD3*
Amplification Peak 39	10q26.11	chr10:120677309-121344955	37	0.016885	46.2	45	
Amplification Peak 40	11q12.1	chr11:56825293-57330723	33	0.024079	38.5	39	
Amplification Peak 41	11q13.4	chr11:74529331-74983324	25	0.016885	46.2	27	
Amplification Peak 42	11q24.2	chr11:124371528-124803387	28	0.016885	46.2	46	
Amplification Peak 43	11q24.2	chr11:125641427-126000192	27	0.016885	53.8	4	
Amplification Peak 44	12p13.33	chr12:2160004-3171873	69	0.016885	46.2	39	
Amplification Peak 45	12q13.13	chr12:52730714-53122322	27	0.019505	46.2	48	
Amplification Peak 46	12q24.13	chr12:111975073-112921584	58	0.024079	46.2	50	
Amplification Peak 47	12q24.22	chr12:115309409-116481375	87	0.016885	46.2	18	
Amplification Peak 48	13q14.3	chr13:48683941-49337754	39	0.024079	38.5	12	
Amplification Peak 49	14q32.31	chr14:98479480-103750044	282	0.016885	46.2	14	*BCL11B*
Amplification Peak 50	16q13	chr16:55439274-56721225	51	0.016885	46.2	14	
Amplification Peak 51	20q13.32	chr20:54421157-56041849	101	0.016885	46.2	45	
Deletion Peak 1	7p14.2	chr7:36428531-37756111	80	0.0011851	53.8	17	
Deletion Peak 2	9q34.2	chr9:135102408-136431045	24	0.0015593	38.5	34	
Deletion Peak 3	13q34	chr13:112554650-114142980	70	0.028348	30.8	25	

Supplementary figures

Figure S1. Location of the probes on X/Y PAR1 region provided in the MLPA probemix and NimbleGen High density microarray platform. X/Y PAR1 region (*CRLF2*, *CSF2RA*, *IL3RA* and *P2RY8*) (5 probes at Xp22.33).

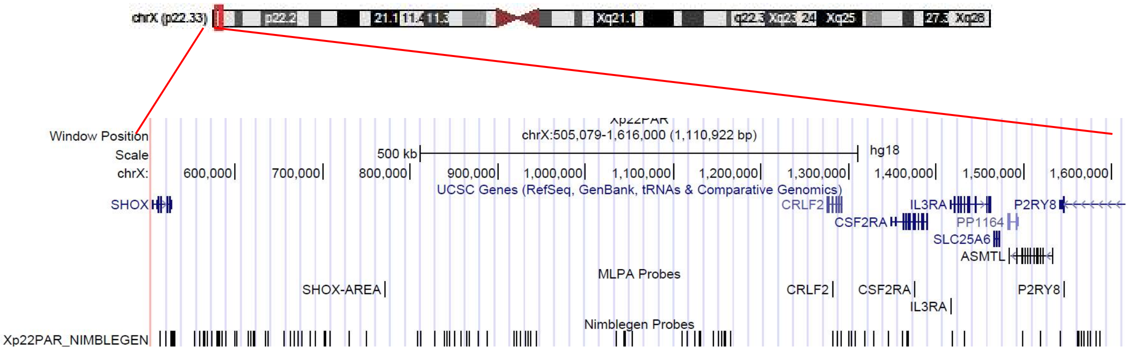

Figure S2. Patterns and frequencies of DNA copy alterations observed in 13 paired diagnostic/relapse samples. A. Frequency of CNAs observed in all samples at diagnosis, B. Frequency of CNAs observed in all samples at relapse. Gains in red and losses in blue.

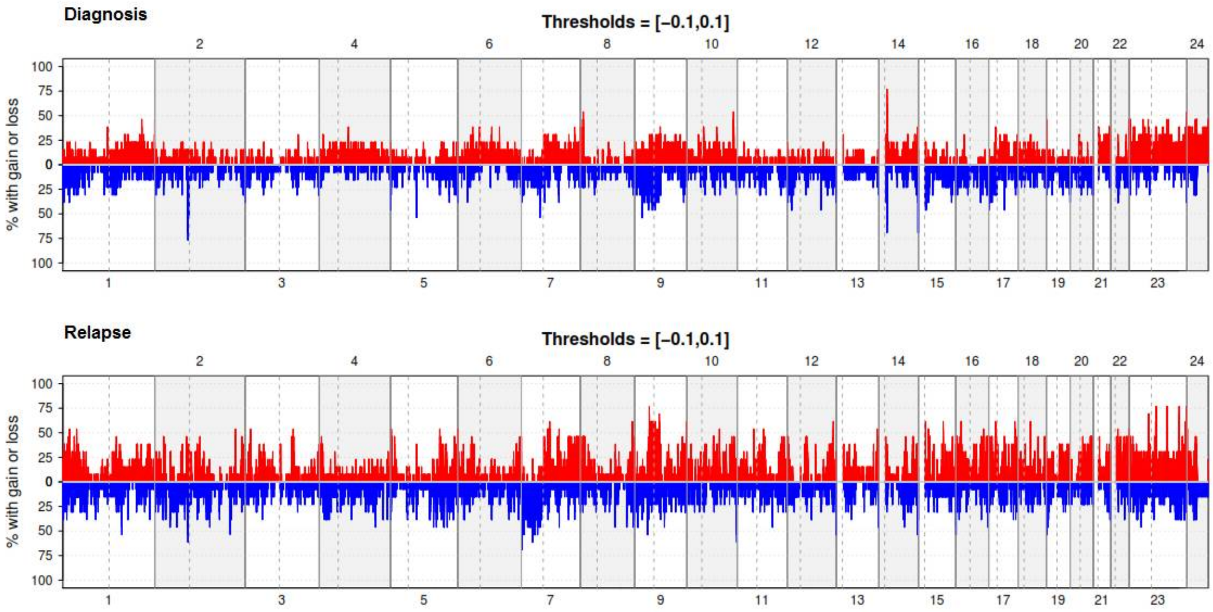

References for supplementary information

1. Beroukhim, R.; Getz, G.; Nghiemphu, L.; Barretina, J.; Hsueh, T.; Linhart, D.; Vivanco, I.; Lee, J.C.; Huang, J.H.; Alexander, S., et al. Assessing the significance of chromosomal aberrations in cancer: methodology and application to glioma. *Proc Natl Acad Sci U S A* **2007**, *104*, 20007-20012, doi:10.1073/pnas.0710052104.
2. Harrison, C.J. Key pathways as therapeutic targets. *Blood* **2011**, *118*, 2935-2936, doi:118/11/2935 [pii]10.1182/blood-2011-07-362723.
3. Chiaretti, S.; Zini, G.; Bassan, R. Diagnosis and subclassification of acute lymphoblastic leukemia. *Mediterranean journal of hematology and infectious diseases* **2014**, *6*, e2014073, doi:10.4084/MJHID.2014.073.
4. Chiaretti, S.; Gianfelici, V.; Ceglie, G.; Foa, R. Genomic characterization of acute leukemias. *Medical principles and practice : international journal of the Kuwait University, Health Science Centre* **2014**, *23*, 487-506, doi:10.1159/000362793.
5. Gowda, C.; Dovat, S. Genetic targets in pediatric acute lymphoblastic leukemia. *Adv Exp Med Biol* **2013**, *779*, 327-340, doi:10.1007/978-1-4614-6176-0_15.
6. Iacobucci, I.; Papayannidis, C.; Lonetti, A.; Ferrari, A.; Baccarani, M.; Martinelli, G. Cytogenetic and molecular predictors of outcome in acute lymphocytic leukemia: recent developments. *Curr Hematol Malig Rep* **2012**, *7*, 133-143, doi:10.1007/s11899-012-0122-5.
7. Loh, M.L.; Mullighan, C.G. Advances in the genetics of high-risk childhood B-progenitor acute lymphoblastic leukemia and juvenile myelomonocytic leukemia: implications for therapy. *Clin Cancer Res* **2012**, *18*, 2754-2767, doi:10.1158/1078-0432.CCR-11-1936.
8. Mullighan, C.G. Genomic profiling of B-progenitor acute lymphoblastic leukemia. *Best Pract Res Clin Haematol* **2011**, *24*, 489-503, doi:S1521-6926(11)00084-3 [pii]10.1016/j.beha.2011.09.004.
9. Roberts, K.G.; Mullighan, C.G. How new advances in genetic analysis are influencing the understanding and treatment of childhood acute leukemia. *Curr Opin Pediatr* **2011**, *23*, 34-40, doi:10.1097/MOP.0b013e3283426260.
10. Woo, J.S.; Alberti, M.O.; Tirado, C.A. Childhood B-acute lymphoblastic leukemia: a genetic update. *Experimental hematology & oncology* **2014**, *3*, 16, doi:10.1186/2162-3619-3-16.
11. Inaba, H.; Greaves, M.; Mullighan, C.G. Acute lymphoblastic leukaemia. *Lancet* **2013**, *381*, 1943-1955, doi:10.1016/S0140-6736(12)62187-4.
12. Pui, C.H.; Carroll, W.L.; Meshinchi, S.; Arceci, R.J. Biology, risk stratification, and therapy of pediatric acute leukemias: an update. *J Clin Oncol* **2011**, *29*, 551-565, doi:JCO.2010.30.7405 [pii]10.1200/JCO.2010.30.7405.
13. Weissmann, S.; Roller, A.; Jeromin, S.; Hernandez, M.; Abaigar, M.; Hernandez-Rivas, J.M.; Grossmann, V.; Haferlach, C.; Kern, W.; Haferlach, T., et al. Prognostic impact and landscape of NOTCH1 mutations in chronic lymphocytic leukemia (CLL): a study on 852 patients. *Leukemia* **2013**, *27*, 2393-2396, doi:10.1038/leu.2013.218.
14. Kohlmann, A.; Klein, H.U.; Weissmann, S.; Bresolin, S.; Chaplin, T.; Cuppens, H.; Haschke-Becher, E.; Garicochea, B.; Grossmann, V.; Hanczaruk, B., et al. The Interlaboratory RObustness of Next-generation sequencing (IRON) study: a deep sequencing investigation of TET2, CBL and KRAS mutations by an international consortium involving 10 laboratories. *Leukemia* **2011**, *25*, 1840-1848, doi:10.1038/leu.2011.155.
15. Grossmann, V.; Roller, A.; Klein, H.U.; Weissmann, S.; Kern, W.; Haferlach, C.; Dugas, M.; Haferlach, T.; Schnittger, S.; Kohlmann, A. Robustness of amplicon deep sequencing underlines its utility in clinical applications. *The Journal of molecular diagnostics : JMD* **2013**, *15*, 473-484, doi:10.1016/j.jmoldx.2013.03.003.
16. Leroy, B.; Anderson, M.; Soussi, T. TP53 mutations in human cancer: database reassessment and prospects for the next decade. *Human mutation* **2014**, *35*, 672-688, doi:10.1002/humu.22552.
17. Schwab, C.J.; Jones, L.R.; Morrison, H.; Ryan, S.L.; Yigittop, H.; Schouten, J.P.; Harrison, C.J. Evaluation of multiplex ligation-dependent probe amplification as a method for the detection of copy number abnormalities in B-cell precursor acute lymphoblastic leukemia. *Genes Chromosomes Cancer* **2010**, *49*, 1104-1113, doi:10.1002/gcc.20818.
